# Exploring AKAPs in visual signaling

**DOI:** 10.3389/fnmol.2024.1412407

**Published:** 2024-05-15

**Authors:** Julia Tomczak, Joanna Mackiewicz, Malwina Lisek, Aleksandra Kaluza, Tomasz Boczek

**Affiliations:** Department of Molecular Neurochemistry, Faculty of Health Sciences, Medical University of Lodz, Lodz, Poland

**Keywords:** A-kinase anchoring proteins, visual system, retina, signaling compartmentalization, retinal ganglion cell

## Abstract

The complex nature of the retina demands well-organized signaling to uphold signal accuracy and avoid interference, a critical aspect in handling a variety of visual stimuli. A-kinase anchoring proteins (AKAPs), known for binding protein kinase A (PKA), contribute to the specificity and efficiency of retinal signaling. They play multifaceted roles in various retinal cell types, influencing photoreceptor sensitivity, neurotransmitter release in bipolar cells, and the integration of visual information in ganglion cells. AKAPs like AKAP79/150 and AKAP95 exhibit distinct subcellular localizations, impacting synaptic transmission and receptor sensitivity in photoreceptors and bipolar cells. Furthermore, AKAPs are involved in neuroprotective mechanisms and axonal degeneration, particularly in retinal ganglion cells. In particular, AKAP6 coordinates stress-specific signaling and promotes neuroprotection following optic nerve injury. As our review underscores the therapeutic potential of targeting AKAP signaling complexes for retinal neuroprotection and enhancement, it acknowledges challenges in developing selective drugs that target complex protein–protein interactions. Overall, this exploration of AKAPs provides valuable insights into the intricacies of retinal signaling, offering a foundation for understanding and potentially addressing retinal disorders.

## 1 Introduction

A-kinase anchoring proteins (AKAPs), named for their role in anchoring signaling molecules, are a diverse family of proteins that share a common feature – the protein kinase A-kinase binding domain (Dodge-Kafka et al., 2005). This domain facilitates the interaction between AKAPs and the regulatory subunits of protein kinase A (PKA), a central player in retinal signaling. This interaction provides a platform for the spatial localization of PKA and other signaling components, allowing for a highly regulated and localized response to extracellular stimuli. The world of AKAPs is characterized by its diversity, both in terms of structure and function. While the A-kinase binding domain is a unifying factor, AKAPs vary widely in their overall structures and associated domains. This diversity allows different AKAPs to engage in distinct protein–protein interactions and participate in various signaling pathways. Some AKAPs, for example, contain domains that interact with ion channels, receptors, or other enzymes, broadening their influence on cellular responses (Dodge-Kafka et al., 2005; Wild and Dell'Acqua, 2018).

Different AKAPs may target PKA to specific subcellular compartments, such as the plasma membrane, mitochondria, or the endoplasmic reticulum (Oliveria et al., 2007; Wild and Dell'Acqua, 2018, 2019; Boczek et al., 2021). This targeted localization ensures that PKA is strategically positioned to phosphorylate substrates in close proximity, optimizing the efficiency of signal transduction. By anchoring PKA and other signaling molecules to discrete locations, AKAPs contribute to the formation of microdomains where signaling events are confined, preventing crosstalk and ensuring that cellular responses are finely tuned and precisely regulated.

The interaction between AKAPs and PKA is central to the functional significance of AKAPs (Kapiloff et al., 1999; Dodge-Kafka et al., 2005). In resting conditions, PKA is bound to the regulatory subunits, maintaining the enzyme in an inactive state. When AKAPs bind to the regulatory subunits, a conformational change occurs, releasing the catalytic subunits and activating PKA. This dynamic regulation ensures that PKA is activated precisely when and where it is needed, contributing to the specificity of cellular responses. AKAP-mediated spatial compartmentalization not only enhances signaling specificity but also allows for dynamic regulation and crosstalk between different signaling pathways. AKAPs can integrate signals from multiple pathways by assembling diverse signaling components in the same microdomain. This dynamic regulation and crosstalk provide cells with the flexibility to respond to a variety of stimuli and adapt to changing environmental conditions.

## 2 The multifaceted roles of AKAPs in different retinal cell types

### 2.1 AKAPs in photoreceptor signaling

Photoreceptor cells, the frontline sensors of the retina, play a pivotal role in the conversion of light into neural signals, forming the foundation of visual perception. AKAPs anchor critical signaling molecules to specific subcellular compartments within photoreceptor cells. This spatial organization allows for localized and precise phosphorylation events, influencing the sensitivity of phototransduction pathways. AKAPs may play a role in modulating the activity of rhodopsin, the light-sensitive protein in rod photoreceptors. By localizing PKA and other effectors, AKAPs contribute to the regulation of rhodopsin phosphorylation, affecting its sensitivity to light and influencing the efficiency of phototransduction. They are also involved in the spatial organization of signaling components associated with light-adaptive mechanisms. This organization ensures that adaptive responses, such as changes in photoreceptor sensitivity, are efficiently initiated and coordinated in response to alterations in ambient light levels. By anchoring signaling molecules to specific microdomains in cone cells, AKAPs participate in the fine-tuning of phototransduction cascades, influencing color discrimination. Beyond their role in signaling, AKAPs may play a role in maintaining the function of cone photoreceptors (Baden et al., 2020).

AKAP79/150 is prominently localized to the synaptic terminals of photoreceptor cells, specifically in the ribbon synapses. This strategic positioning enables it to influence synaptic transmission by anchoring protein kinase A (PKA) and other signaling effectors. AKAP79/150 plays a crucial role in modulating the phosphorylation status of synaptic proteins, influencing neurotransmitter release. Its presence in photoreceptor terminals highlights its significance in shaping the initial stages of visual signal processing (Wang and Cortes, 2021).

AKAP450 is found in the inner segments of photoreceptor cells, adjacent to the connecting cilium. Its subcellular localization suggests a role in the regulation of processes occurring in this crucial region. The subcellular positioning of AKAP450 hints at its involvement in processes such as ciliogenesis. By influencing events in the inner segments, AKAP450 may contribute to the maintenance of photoreceptor structure and function (Subramanian and Sahoo, 2022).

### 2.2 AKAPs in bipolar cell signaling

In the complex landscape of retinal circuits, bipolar cells stand as pivotal intermediaries, translating signals from photoreceptors into meaningful neural information. AKAPs contribute to the fine-tuning of neurotransmitter release from bipolar cells (Bastola et al., 2023). By anchoring PKA and other effectors, AKAPs modulate the activity of calcium channels and vesicle release, influencing the strength and timing of synaptic transmission. AKAPs anchor signaling components in close proximity to receptors on bipolar cell dendrites. This spatial arrangement may influence the responsiveness of bipolar cells to signals received from photoreceptors, contributing to the modulation of receptor sensitivity. AKAPs also facilitate the integration of various signaling pathways. AKAP-mediated spatial organization contributes to the diversity of responses observed in different bipolar cell subtypes (Baden et al., 2020).

Dysregulation of AKAP-mediated signaling in bipolar cells may contribute to visual disorders affecting synaptic transmission. Investigating these mechanisms can provide insights into conditions, where altered signaling in bipolar cells plays a role, such as bipolar cell-associated retinal dystrophies. AKAP95 is an example of an anchoring protein found in bipolar cell dendrites, positioned near glutamate receptors. This subcellular localization allows AKAP95 to regulate receptor sensitivity and influence the responsiveness of bipolar cells to signals from photoreceptors. By tethering PKA and other effectors, AKAP95 contributes to the fine-tuning of receptor sensitivity, ensuring that bipolar cells can efficiently process and modulate signals before transmission to downstream neurons (Roa et al., 2021).

### 2.3 AKAPs in ganglion cells

AKAP150 is found in ganglion cells, positioning itself in the vicinity of ion channels and receptors crucial for signal transmission. This localization allows AKAP150 to influence the integration of signals within ganglion cells before they are transmitted to the brain. AKAP150 plays a key role in the integration of signals within ganglion cells, facilitating the convergence of inputs from various bipolar cells. Its presence near critical signaling components contributes to the orchestration of diverse visual information (Ostroveanu et al., 2007). Beyond their role in signaling, AKAPs may be involved in neuroprotective mechanisms within ganglion cells (Boczek et al., 2019; Boczek and Kapiloff, 2020; Bastola et al., 2023). Their roles in regulating cellular processes associated with survival highlight the potential of AKAPs as key players in preservation of ganglion cell function.

**Figure 1:**
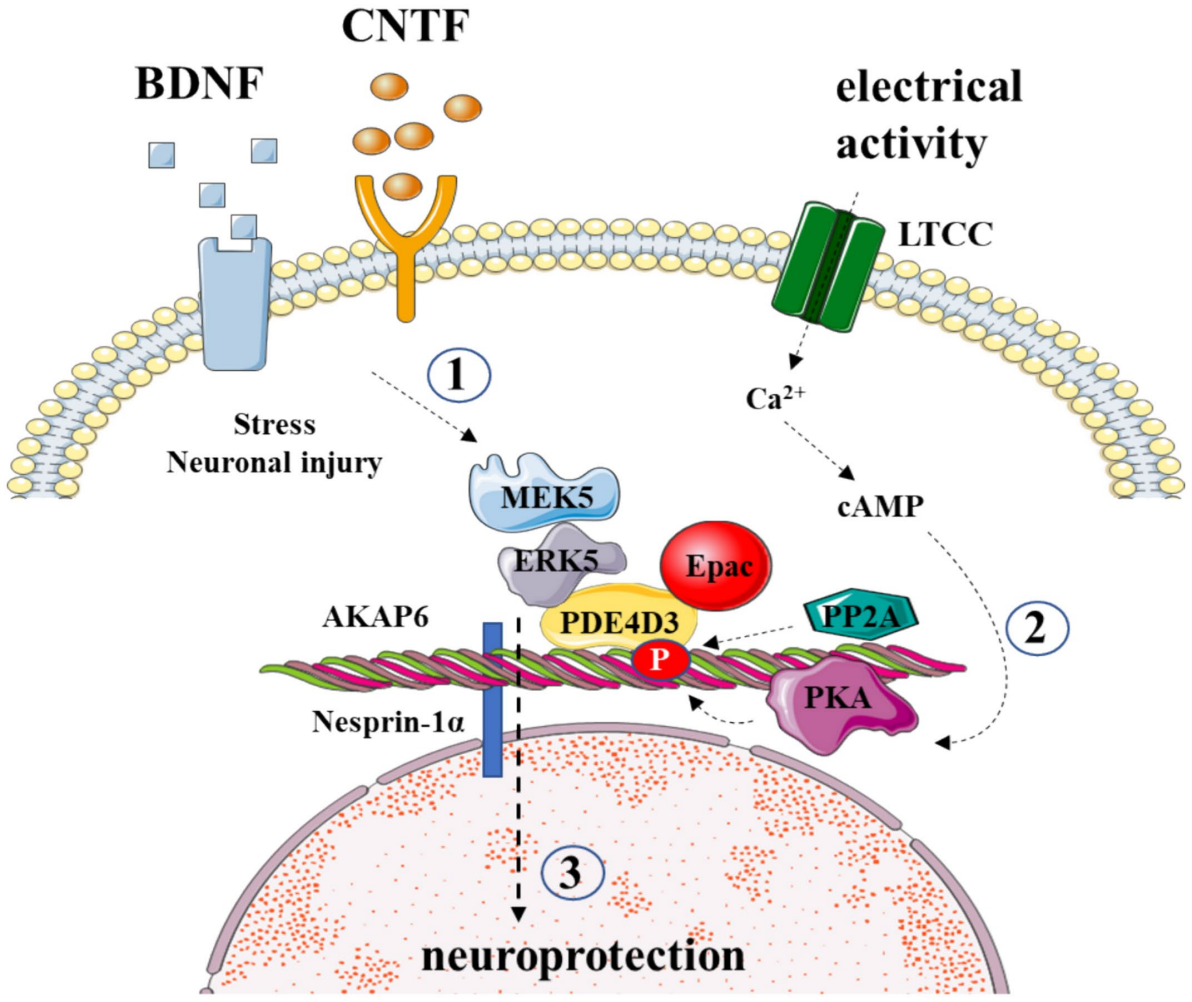
AKAP6 orchestrates the survival of retinal ganglion cells post-injury. (1) AKAP6 binds to PDE4D3, securing ERK5-MAPK pathway signaling elements, which respond to neurotrophic signaling; (2) AKAP6 tethers PKA, responsive to electrical activity; (3) the crosstalk of these pathways confers RGC survival after injury.

Recently, we have demonstrated that AKAP6-mediated signaling (Figure 1) in critical for survival of retinal ganglion cells (Boczek et al., 2019). By using genetically engineered delocalizing peptides, we confirmed that local cAMP/PKA pro-survival signaling within the AKAP6 signalosome is mediated by anchored phosphodiesterase 4D3 activity and enhanced ganglion cell survival can be achieved by increasing perinuclear cAMP concentration (Boczek et al., 2019). Furthermore, we have also developed a FRET-based technique to measure PKA signaling activity within AKAP6 signalosome that can be applied both *in vitro* and *in vivo* and help to monitor the changes in cAMP signaling following optic nerve injury. The expression of AKAPs in a particular cell type of retina, along with their binding partners, is summarized in Table 1.

**Table 1:** Expression of AKAPs in retinal cell types along with their potential binding partners.

AKAP	Expression	Potential proteins in complex
AKAP79/150	Photoreceptor cell ganglion cells	PKA (Coghlan et al., 1995), Protein kinase C (Coghlan et al., 1995), PP2B (Klauck et al., 1996), N-methyl-D-aspartate receptor (Colledge et al., 2000), α-Amino-3-hydroxy-5-methyl-4-isoxazolepropionic (AMPA) receptor (Colledge et al., 2000), postsynaptic protein of 95 kDa (PSD-95) (Colledge et al., 2000), synapse-associated protein-97 (SAP97) (Colledge et al., 2000), KCNQ2 channel (Hoshi et al., 2003), L-type voltage-gated Ca^2+^ channels (Gao et al., 1997), aquaporin water channels (Jo et al., 2001)
AKAP450	Photoreceptor cells	PKA (Westphal et al., 1999), PP1 (Westphal et al., 1999), NMDA receptor (Westphal et al., 1999), KCNQ1channel (Marx et al., 2002), Inositol-1,4,5-trisphosphate receptor (Tu et al., 2004), Protein kinase Cε (Takahashi et al., 2000), Protein kinase N (Takahashi et al., 1999), Casein kinase-1 (Sillibourne et al., 2002), PP2A (Takahashi et al., 1999), Intracellular Cl^−^ channels (CLIC) (Shanks et al., 2002), γ-tubulin-complex protein-2 and − 3 (Takahashi et al., 2002), PDE4D3 (Taskén et al., 2001)
AKAP95	Bipolar cells	PKA (Asirvatham et al., 2004), p68 RNA helicase (Akileswaran et al., 2001), D-type Cyclins (Arsenijevic et al., 2004), PDE4A (Asirvatham et al., 2004), AMY-1 (Furusawa et al., 2002), ACAPD2/Eg7 (Steen et al., 2000), caspase-3 (Kamada et al., 2005), G1-S Cyclins (Arsenijevic et al., 2006), fidgetin (Yang et al., 2006), HDAC3 (Dirksen et al., 2006)
AKAP6	Ganglion cells	PKA (McCartney et al., 1995), PDE4D3 (Dodge et al., 2001), RyR (Marx et al., 2000; Kapiloff et al., 2001), PP1 (Marx et al., 2000), NCX1 (Schulze et al., 2003), PKC (Schulze et al., 2003), PP2A (Marx et al., 2000), nesprin-1α (Pare et al., 2005), Epac1 (Dodge-Kafka et al., 2005), ERK5-kinase (Dodge-Kafka et al., 2005), PDK1 (Michel et al., 2005)

## 3 AKAP in axonal degeneration in the retina

The insufficient trophic signaling has long been implicated as the cause of CNS neuronal regeneration failure (Goldberg and Barres, 2000). Notably, the application of neurotrophic factors such as brain-derived neurotrophic factor (BDNF), glial-derived neurotrophic factor (GDNF), ciliary neurotrophic factor (CNTF), and fibroblast growth factor (FGF) can delay the death of retinal ganglion cells (RGCs) (Unoki and LaVail, 1994; Mo et al., 2002). Trophic signaling can be enhanced by electrical activity and cAMP elevations, providing neuroprotection (Corredor et al., 2012). The inhibition of PKA eliminates these protective effects, indicating a potential role for AKAP scaffolding (Goldberg et al., 2002). Recent research implicates muscle AKAP (mAKAP) in coordinating stress-specific signaling and axon growth in RGCs (Wang et al., 2015). mAKAP, with its three spectrin repeats, localizes to the outer nuclear membrane through interaction with the protein nesprin-1α. Two splice variants, mAKAPα and mAKAPβ, show tissue-specific expression patterns (Kapiloff et al., 1999; Dodge-Kafka et al., 2005). While mAKAPβ assembles signaling complexes in cardiomyocytes, mAKAPα, predominantly expressed in the brain, contains an additional anchoring site for 3-phosphoinositide-dependent kinase-1 (PDK1) (Michel et al., 2005).

Wang et al.’s study highlights the expression of mAKAPα in retinal ganglion cells and its crucial role in transducing pro-survival signaling induced by cAMP, BDNF, and CNTF. Knockout of mAKAPα hinders trophic signaling from promoting survival after optic nerve injury (Wang et al., 2015). Although the specific mechanisms of mAKAPα-directed neuroprotective signaling remain unclear, it is plausible that the scaffold facilitates crosstalk between cAMP and MAPK pathways. mAKAPα-anchored PKA and PP2A positively and negatively modulate PDE4D3 activity, respectively, controlling the local cAMP concentration at the ONM. Additionally, ERK5 indirectly binds to mAKAPα through association with PDE4D3. While further studies are required to elucidate the exact cross-talk mechanisms, it is evident that mAKAPα signaling plays a central role in the neuroprotective effects of neurotrophins following RGC damage. A more comprehensive understanding of these processes could pave the way for the development of neuroprotective interventions after neuronal injury and degeneration.

## 4 Targeting AKAPs as a therapeutic strategy in retinal degeneration

Disrupting AKAP signaling complexes to influence pathological signaling holds significant promise, particularly through the uncoupling of AKAP signaling components, allowing for targeted disruption within spatially confined nanodomains. With different AKAPs present in each cell type, each with distinct subcellular locations and functions (Kennedy and Scott, 2015), a key objective of future research is the development of agents that selectively target protein–protein interactions (PPIs) between specific AKAP isoforms and their binding partners. Currently, three classes of molecules—peptides, peptidomimetics, and small molecule inhibitors—are employed to disrupt AKAP-PPIs.

The initial tool developed to disrupt the RII-AKAP binding was a 24-amino acid peptide named Ht31, derived from the PKA binding domain of AKAP-Lbc (Carr et al., 1992). While Ht31 binds to all R subunit isoforms of PKA, it exhibits a higher affinity for RII and has been employed as a non-selective blocker of all AKAP-PKA interactions. Subsequent advancements have led to the development of more selective peptides with increased affinity for either RI or RII subunits. Notably, RIAD demonstrates a 1,000-fold higher affinity for RI, while superAKAP-IS exhibits a > 10,000-fold higher affinity for RII (Gold et al., 2006). Despite the utility of peptides in studying AKAP function in the nervous system by disrupting AKAP-PKA interactions, their instability within the cell and the potential for membrane retention, particularly with peptides like St-Ht31, place constraints on their therapeutic applicability (Kennedy and Scott, 2015).

Efforts have been made to improve the stability and permeability of peptides, leading to the emergence of peptidomimetics. This innovative category of inhibitors undergoes chemical modification with hydrocarbon positioned along the peptide backbone, accomplished by introducing non-natural amino acids into the peptide sequence, followed by chemical cross-linking (Wang et al., 2014). The result is a peptide with a structurally constrained α-helical configuration, termed a Stapled Anchoring Disruptor (STAD). Importantly, these peptidomimetics demonstrate significantly enhanced membrane permeability and intracellular stability (Kennedy and Scott, 2015; Hanold et al., 2017). Isoform-selective STADs have been effectively crafted to target the AKAP binding pocket on both the RI- and RII-subunit D/D domain (Wang et al., 2014). Although the current applicability of these proteins is constrained, this strategy is poised to inform the future development of compounds targeted at AKAP.

From a therapeutic perspective, small molecule inhibitors present several advantages compared to larger peptide molecules, such as heightened oral bioavailability, enhanced intracellular stability, and cost-effectiveness. Nonetheless, the design of small molecules targeting protein–protein interactions comes with added challenges, given the typically expansive and discontinuous surface area involved in these interactions, encompassing multiple essential residues (Calejo and Taskén, 2015). The inaugural small molecule inhibitor of PKA, FMP-API-1, was introduced, binding to a site external to the D/D domain and exerting allosteric inhibition of the AKAP-PKA interaction (Christian et al., 2011). Originating from high-throughput screening, this molecule disrupted the binding of both RI or RII to AKAP18δ, although the precise mechanism remains incompletely elucidated. The allosteric effects also prompted PKA activation, potentially through interaction with the auto-inhibitory domain. Consequently, further refinement of the compound is deemed necessary (Christian et al., 2011).

## 5 Conclusion

The continuous quest for innovative drug targets is focused on selectively modifying pathologically active signaling pathways with subcellular precision. The goal of this approach is to develop compounds with fewer off-target effects in comparison to those designed for broadly expressed signaling molecules such as kinases, phosphatases, ion channels, and receptors. AKAP-mediated signal compartmentalization is crucial for the nervous system, as it co-localizes signaling molecules within nanodomain compartments, orchestrating various critical processes known to be disrupted in injured retina. As a result, the acknowledgment of the therapeutic potential of targeting AKAP signaling complexes has gained momentum.

This review delves into the engagement of AKAPs in retinal functions. A growing body of evidence suggests that many insults leading to the degeneration of retinal ganglion cells could either be a consequence of AKAP dysfunction or have the potential to be alleviated through the modification of AKAP interactions. However, conclusive proof-of-principle studies utilizing genetic manipulations (e.g., knockout, knock-in, RNAi approaches) to disrupt AKAP signaling functions in animal disease models are essential to further validate AKAPs as viable therapeutic targets for retinal neuroprotection and neuroenhancement. A significant challenge in AKAP-centered drug discovery lies in the development of stable drugs capable of targeting complex protein–protein interactions between individual AKAPs and their binding partners with high selectivity. Despite efforts to generate peptides and small molecules for understanding the role of AKAP-anchored enzymes in neuronal physiology and disease, their therapeutic utility is currently limited.

## Author contributions

JT: Conceptualization, Investigation, Software, Visualization, Writing – original draft, Writing – review & editing. JM: Conceptualization, Investigation, Resources, Visualization, Writing – original draft, Writing – review & editing. ML: Conceptualization, Data curation, Investigation, Resources, Software, Supervision, Visualization, Writing – original draft, Writing – review & editing. AK: Conceptualization, Investigation, Visualization, Writing – original draft, Writing – review & editing. TB: Conceptualization, Funding acquisition, Investigation, Project administration, Resources, Software, Visualization, Writing – original draft, Writing – review & editing.
